# The chromosomal genome sequence of a staghorn coral, *Acropora* cf. *manni* (Scleractinia: Acroporidae)

**DOI:** 10.12688/wellcomeopenres.27174.1

**Published:** 2026-08-27

**Authors:** David J Combosch, Andrew H Baird, David R Burdick, Hector Torrado, Dareon Rios, Sebastian Metz, Michael Sweet, Graeme Oatley, Elizabeth Sinclair, Eerik Aunin, Noah Gettle, Camilla Santos, Michael Paulini, Haoyu Niu, Victoria McKenna, Rebecca O’Brien, Wellcome Sanger Institute Tree of Life Management, Samples and Laboratory team, Wellcome Sanger Institute Scientific Operations: Sequencing Operations, Wellcome Sanger Institute Tree of Life Core Informatics team, EBI Aquatic Symbiosis Genomics Data Portal Team, Aquatic Symbiosis Genomics Project Leadership

**Affiliations:** 1 University of Guam, Marine Laboratory, Mangilao, Guam, USA; 2 College of Science and Engineering, James Cook University, Townsville, Queensland, Australia; 3 Aquatic Research Facility, Nature-based Solutions Research Centre, University of Derby, Derby, England, UK; 4 Tree of Life Programme, Wellcome Sanger Institute, Hinxton, England, UK

**Keywords:** Acropora cf. manni, staghorn coral, genome sequence, chromosomal, Scleractinia, microbial metagenome assembly

## Abstract

We present a genome assembly from a colony sample of *Acropora cf. manni* (staghorn coral; Cnidaria; Anthozoa; Scleractinia; Acroporidae). The assembly contains two haplotypes with total lengths of 459.26 megabases and 489.30 megabases. Most of haplotype 1 (94.39%) is scaffolded into 14 chromosomal pseudomolecules. Haplotype 2 was assembled to scaffold level. The mitochondrial genome has also been assembled, with a length of 18.48 kilobases. Metagenome binning recovered one bacterial bin from the phylum Chlamydiota.

## Species taxonomy

Eukaryota; Opisthokonta; Metazoa; Eumetazoa; Cnidaria; Anthozoa; Hexacorallia; Scleractinia; Refertina; Acroporidae; *Acropora*; *Acropora cf. manni* (NCBI:txid3471590).

## Background

*Acropora* Oken, 1815 belongs to the family Acroporidae and is the most species-rich and widespread reef-building coral genus. It contains 407 nominal species ( Hoeksema & Cairns, 2025) and is currently in significant taxonomic flux ( Bridge *et al.*, 2024; Rassmussen *et al.*, 2025). The genus is distributed from the western Indian Ocean to the Caribbean, although its presence in Hawaii and the tropical eastern Pacific may be ephemeral, and from Lord Howe Island to southern Japan, including the Red Sea and temperate coral regions ( Veron *et al.*, 2015). *Acropora* corals often dominate shallow coral assemblages, from lagoons and reef flats to exposed fore reefs, but tend to be less abundant below 30 m. The genus is a major contributor to reef accretion and structural complexity, but many species are susceptible to thermal stress, predation by crown-of-thorns seastars and coral disease, and substantial mortality has been recorded worldwide over the past four decades ( Cramer *et al.*, 2020; De’ath *et al.*, 2012; Manzello *et al.*, 2025; Marshall & Baird, 2000).

Staghorn *Acropora* corals are important habitat-forming species in many back-reef and lagoon habitats. On Guam and across the Mariana Islands, *Acropora* cf. *manni* is the most abundant staghorn coral and dominates many back-reef habitats, especially along the calmer western shore of Guam. Following recent bleaching and low-tide-related coral mortalities, a coral restoration programme has been established on Guam, with a focus on staghorn *Acropora* corals, especially *Acropora* cf. *manni* ( Raymundo *et al.*, 2022).

This has led to increased local research on this species, mostly under the names *Acropora pulchra* and *Acropora* cf. *pulchra.* Studies have examined its growth and morphology, reproductive biology, symbiosis, population genetics and restoration ( Fifer *et al.*, 2021; Goldman, 2007; Lapacek, 2017; Miller, 2023; Raymundo *et al.*, 2022; Rios, 2020; Rios *et al.*, 2025; Torrado *et al.*, 2025). Rios et al. (2025) conducted a population genomic study to inform restoration and found large clonal clusters, very low levels of genetic diversity and isolation by distance over tens of kilometres. Torrado et al. (2025) also included this species in a comparative genomic study of two co-occurring fore-reef coral species on Guam. The reproductive biology of this coral was investigated in an MSc thesis at the University of Guam, with spawning observed in April and May 2016 and 2017, two to seven days after the full moon ( Lapacek, 2017).

The Guam staghorn *Acropora* represented by this specimen has a complex taxonomic history. Earlier records identified similar material from Guam as *Acropora aspera* and noted its morphological similarity to *A. pulchra* and *A. exigua*; Mariana material was distinguished by the poorly developed first cycle of septa in the radial corallites ( Randall, 2003; Randall & Myers, 1983). The Guam voucher differs from the holotype of *A. pulchra*, which has tubular radial corallites with oblique openings, whereas the Guam voucher has labellate radial corallites with square lips. Its morphology is closer to that of *A. manni* (Quelch, 1886), described from the Philippines, and we therefore refer to the species here as *Acropora* cf. *manni.* A further possible name is *A. cribripora* (Dana, 1846), although both *A. manni* and *A. cribripora* are currently treated as junior synonyms of *A. aspera* ( Hoeksema & Cairns, 2025; Wallace, 1978).

As part of the Aquatic Symbiosis Genomics Project, we sequenced the genome of *Acropora* cf. *manni* (specimen jaAcrPulc1). This genome provides a reference for studies of staghorn *Acropora* corals in Guam and the Mariana Islands, including work on taxonomy, symbiosis, population structure and reef restoration.

## Methods

### Sample acquisition

**Figure 1.**
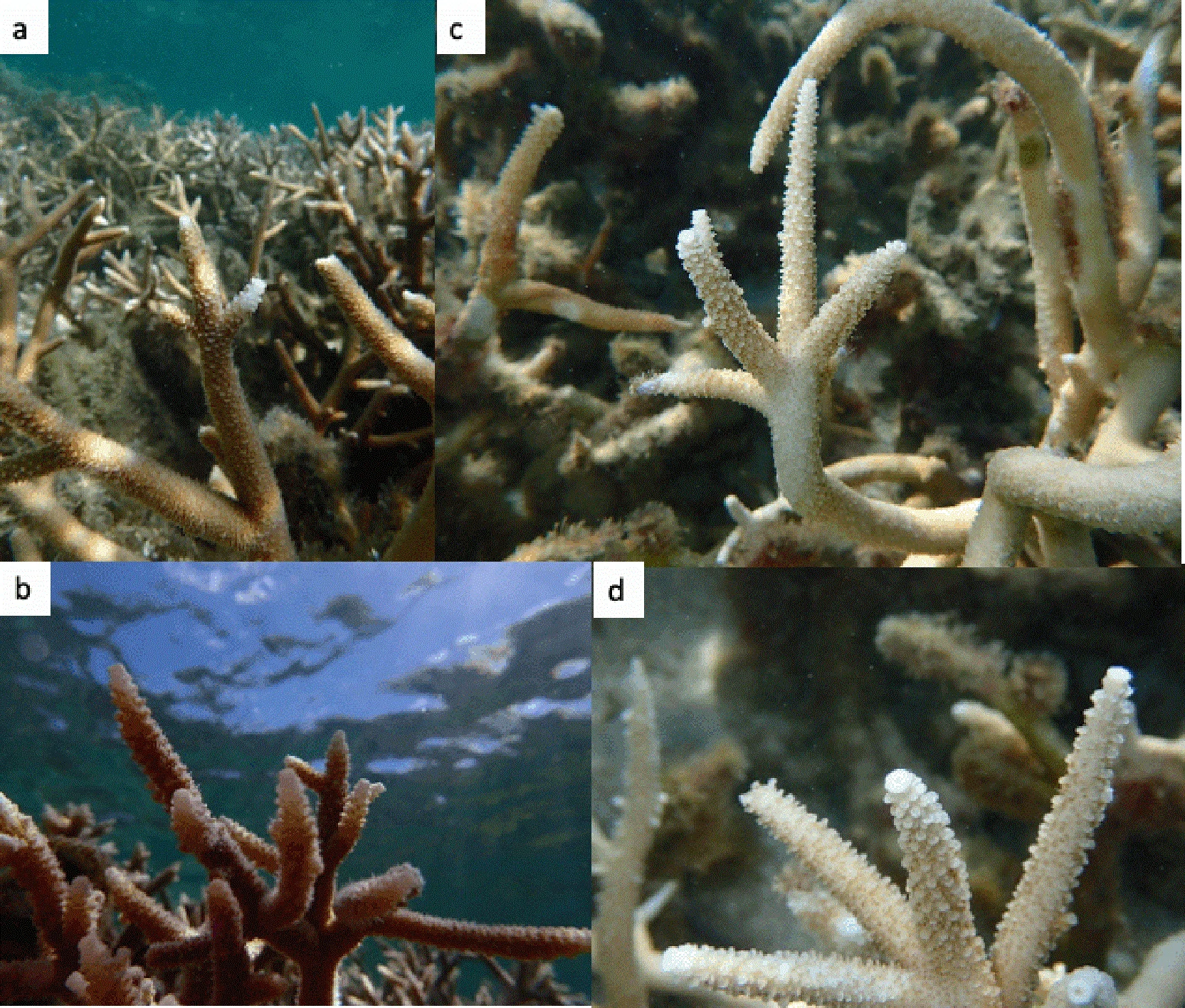
Images of the *Acropora* cf. *manni* (jaAcrPulc1) specimen used for genome sequencing.

A colony sample consisting of multiple branches of an adult *A.* cf. *manni* colony (specimen ID UDUK0000426, ToLID jaAcrPulc1; Figure 1) was collected from a shallow back reef habitat from Aniguk/West Agaña Bay, in the central part of Guam, Mariana Islands (latitude 13.479806, longitude 144.741583) on 2021-05-24. The specimen was identified and transported alive to the UOG Marine Laboratory where it was processed using wire cutters. Multiple tissue samples were preserved in 2 ml DESS and stored at −20 °C until shipping. The sampling adhered to relevant research protocols and regulatory guidelines ( Voolstra *et al.*, 2021), for the responsible use of coral resources for scientific inquiry. The skeletal voucher is stored at the University of Guam Biorepository on Guam. The specimen was collected by David Combosch (University of Guam) and identified by David Combosch and Andrew Baird (James Cook University).

### Taxonomic information

Unpublished molecular analyses using the clade scheme of Cowman *et al*. (2020) indicate that *A. aspera* belongs to clade III and *A. pulchra* to clade V, whereas the Guam genome and specimens similar to the holotype of *A. manni* fall in clade VI. Future work including topotypes of *A. manni*, *A. cribripora*, *A. pulchra* and *A. aspera* will be needed to confirm the identification of this sample. The voucher is also likely to represent a different species from the putative *A. pulchra* genome on NCBI (GenBank assembly GCA_044231415), which was generated from a specimen from Moorea, French Polynesia ( Conn *et al.*, 2025).

Available records suggest that *Acropora* cf. *manni* occurs in the western and central Pacific, including Guam, the Philippines, Japan and Fiji. In the Mariana Islands, it is a hermaphroditic broadcast spawner ( Lapacek, 2017). Its symbiotic associations in Guam are dominated by members of the Symbiodiniaceae in the genera *Cladocopium* and *Durusdinium*, mainly *Cladocopium* C40, C3 and C116 or *Durusdinium* D1 ( Rios *et al.*, 2025).

### Nucleic acid extraction

Protocols for high molecular weight (HMW) DNA extraction developed at the Wellcome Sanger Institute (WSI) Tree of Life Core Laboratory are available on protocols.io ( Howard *et al.*, 2025). The jaAcrPulc1 sample was weighed and triaged to determine the appropriate extraction protocol.

Tissue was disrupted using the bead beating protocol. HMW DNA was extracted using the Manual MagAttract protocol. We used centrifuge-mediated fragmentation to produce DNA fragments in the 8–10 kb range, following the Covaris g-TUBE protocol for ultra-low input (ULI). Sheared DNA was purified by automated SPRI (solid-phase reversible immobilisation).

The concentration of the sheared and purified DNA was assessed using a Nanodrop spectrophotometer and Qubit Fluorometer using the Qubit dsDNA High Sensitivity Assay kit. Fragment size distribution was evaluated by running the sample on the FemtoPulse system. For this sample, the final post-shearing DNA had a Qubit concentration of 1.83 ng/μL, with a fragment size of 6.3 kb.

RNA was extracted from the jaAcrPulc1 specimen in the Tree of Life Laboratory at the WSI using the RNA Extraction: Automated MagMax™ *mir*Vana protocol. The RNA concentration was assessed using a Nanodrop spectrophotometer and a Qubit Fluorometer using the Qubit RNA Broad-Range Assay kit. Analysis of the integrity of the RNA was done using the Agilent RNA 6000 Pico Kit and Eukaryotic Total RNA assay.

### PacBio HiFi library preparation and sequencing

Library preparation and sequencing were performed at the WSI Scientific Operations core. Prior to library preparation, the DNA was fragmented to ~10 kb. Ultra-low-input (ULI) libraries were prepared using the PacBio SMRTbell® Express Template Prep Kit 2.0 and gDNA Sample Amplification Kit. Samples were normalised to 20 ng DNA. Single-strand overhang removal, DNA damage repair, and end-repair/A-tailing were performed according to the manufacturer’s instructions, followed by adapter ligation. A 0.85× pre-PCR clean-up was carried out with Promega ProNex beads.

The DNA was evenly divided into two aliquots for dual PCR (reactions A and B), both following the manufacturer’s protocol. A 0.85× post-PCR clean-up was performed with ProNex beads. DNA concentration was measured using a Qubit Fluorometer v4.0 (Thermo Fisher Scientific) with the Qubit HS Assay Kit, and fragment size was assessed on an Agilent Femto Pulse Automated Pulsed Field CE Instrument (Agilent Technologies) using the gDNA 55 kb BAC analysis kit. PCR reactions A and B were then pooled, ensuring a total mass of ≥500 ng in 47.4 μl.

The pooled sample underwent another round of DNA damage repair, end-repair/A-tailing, and hairpin adapter ligation. A 1× clean-up was performed with ProNex beads, followed by DNA quantification using the Qubit and fragment size analysis using the Agilent Femto Pulse. Size selection was performed on the Sage Sciences PippinHT system, with target fragment size determined by Femto Pulse analysis (typically 4–9 kb). Size-selected libraries were cleaned with 1.0× ProNex beads and normalised to 2 nM before sequencing.

The sample was sequenced on a Revio instrument (Pacific Biosciences, California, USA). Prepared libraries with a molarity greater than 2 nM were normalised to 2 nM, and 15 μL was used for making complexes. For libraries with a molarity below 2 nM, the available 10 μL library volume was used without normalisation. Primers were annealed and polymerases were bound to generate circularised complexes, following the manufacturer’s instructions. Complexes were purified using 1.2× SMRTbell beads, diluted to the Revio loading concentration of 200–300 pM, and spiked with a Revio sequencing internal control. The sample was sequenced on a Revio 25 M SMRT cell. SMRT Link software (Pacific Biosciences), a web-based workflow manager, was used to configure and monitor the run and to carry out primary and secondary data analysis.

### Hi-C

***Sample preparation and crosslinking***

The Hi-C sample was prepared from 20–50 mg of frozen tissue from the jaAcrPulc1 sample using the Arima-HiC v2 kit (Arima Genomics). Following the manufacturer’s instructions, tissue was fixed and DNA crosslinked using TC buffer to a final formaldehyde concentration of 2%. The tissue was homogenised using the Diagnocine Power Masher-II. Crosslinked DNA was digested with a restriction enzyme master mix, biotinylated, and ligated. Clean-up was performed with SPRISelect beads before library preparation. DNA concentration was measured with the Qubit Fluorometer (Thermo Fisher Scientific) and Qubit HS Assay Kit. The biotinylation percentage was estimated using the Arima-HiC v2 QC beads.

***Hi-C library preparation and sequencing***

Biotinylated DNA constructs were fragmented using a Covaris E220 sonicator and size selected to 400–600 bp using SPRISelect beads. DNA was enriched with Arima-HiC v2 kit Enrichment beads. End repair, A-tailing, and adapter ligation were carried out with the NEBNext Ultra II DNA Library Prep Kit (New England Biolabs), following a modified protocol where library preparation occurs while DNA remains bound to the Enrichment beads. Library amplification was performed using KAPA HiFi HotStart mix and a custom Unique Dual Index (UDI) barcode set (Integrated DNA Technologies). Depending on sample concentration and biotinylation percentage determined at the crosslinking stage, libraries were amplified with 10–16 PCR cycles. Post-PCR clean-up was performed with SPRISelect beads. Libraries were quantified using the AccuClear Ultra High Sensitivity dsDNA Standards Assay Kit (Biotium) and a FLUOstar Omega plate reader (BMG Labtech).

Prior to sequencing, libraries were normalised to 10 ng/μL. Normalised libraries were quantified again to create equimolar and/or weighted 2.8 nM pools. Pool concentrations were checked using the Agilent 4200 TapeStation (Agilent) with High Sensitivity D500 reagents before sequencing. Sequencing was performed using paired-end 150 bp reads on the Illumina NovaSeq X.

### RNA library preparation and sequencing

Libraries were prepared using the NEBNext® Ultra II Directional RNA Library Prep Kit for Illumina (New England Biolabs), following the manufacturer’s instructions. Poly(A) mRNA was isolated from 100 ng of total RNA using oligo (dT) beads, converted to cDNA, and uniquely indexed; 14 PCR cycles were performed. Libraries were prepared with an intended fragment size of 100–300 bp. Libraries were quantified, normalised, and pooled equimolarly to a final concentration of 2.8 nM before dilution to 150 pM for loading. Sequencing was carried out on the Illumina NovaSeq X, generating paired-end reads.

### Genome assembly

Prior to assembly of the PacBio HiFi reads, a database of *k*-mer counts ( *k* = 31) was generated from the filtered reads using FastK. GenomeScope2 ( Ranallo-Benavidez *et al.*, 2020) was used to analyse the *k*-mer frequency distributions, providing estimates of genome size, heterozygosity, and repeat content.

The HiFi reads were assembled using Hifiasm in Hi-C phasing mode ( Cheng *et al.*, 2021, 2022), producing two haplotypes. Hi-C reads ( Rao *et al.*, 2014) were mapped to the primary contigs using bwa-mem2 ( Vasimuddin *et al.*, 2019). Contigs were further scaffolded with Hi-C data in YaHS ( Zhou *et al.*, 2023), using the --break option for handling potential misassemblies. The scaffolded assemblies were evaluated using Gfastats ( Formenti *et al.*, 2022), BUSCO ( Manni *et al.*, 2021) and MerquryFK ( Rhie *et al.*, 2020).

The mitochondrial genome was assembled using MitoHiFi ( Uliano-Silva *et al.*, 2023) and OATK ( Zhou *et al.*, 2025).

### Assembly curation

The assembly was decontaminated using the Assembly Screen for Cobionts and Contaminants ( ASCC) pipeline. TreeVal was used to generate the flat files and maps for use in curation. Manual curation was conducted primarily in PretextView and HiGlass ( Kerpedjiev *et al.*, 2018). Scaffolds were visually inspected and corrected as described by Howe *et al*. (2021). Manual corrections included 199 breaks, 223 joins, and removal of 130 haplotypic duplications. This reduced the scaffold count by 11.7%, increased the scaffold N50 by 164.2%, and reduced the total assembly length by 4.9%. The curation process is described at sanger-tol/curation-resources . PretextSnapshot was used to generate a Hi-C contact map of the final assembly.

### Assembly quality assessment

The Merqury.FK tool ( Rhie *et al.*, 2020) was run in a Singularity container ( Kurtzer *et al.*, 2017) to evaluate *k*-mer completeness and assembly quality for both haplotypes using the *k*-mer database ( *k* = 31) computed prior to genome assembly. The analysis outputs included assembly QV scores and completeness statistics.

The genome was analysed using the BlobToolKit pipeline, a Nextflow implementation of the earlier Snakemake version ( Challis *et al.*, 2020). PacBio reads were aligned to the assembly using minimap2 ( Li, 2018) and processed with SAMtools ( Danecek *et al.*, 2021) to generate coverage tracks. The pipeline runs BUSCO ( Manni *et al.*, 2021) using lineages identified from the NCBI Taxonomy ( Schoch *et al.*, 2020). For the three basal BUSCO lineages, eukaryota, bacteria and archaea, BUSCO genes are aligned to the UniProt Reference Proteomes database ( Bateman *et al.*, 2023) using DIAMOND BLASTp ( Buchfink *et al.*, 2021). The genome assembly is divided into chunks based on the density of BUSCO genes from the closest taxonomic lineage, and each chunk is aligned to the UniProt Reference Proteomes database using DIAMOND BLASTx. Sequences without DIAMOND BLASTx hits are extracted using seqtk and aligned to the NT database using BLASTn ( Altschul *et al.*, 1990). The outputs are consolidated into a BlobDir for visualisation in BlobToolKit. The BlobToolKit pipeline was developed using nf-core tooling ( Ewels *et al.*, 2020) and MultiQC ( Ewels *et al.*, 2016), with containerisation through Docker ( Merkel, 2014) and Singularity ( Kurtzer *et al.*, 2017).

### Metagenome assembly

A metagenome assembly was generated using MetaMDBG ( Benoit *et al.*, 2024), and the recovered bin was refined using MAGScoT ( Rühlemann *et al.*, 2022). PROKKA ( Seemann, 2014) was used to identify tRNA and rRNA genes, CheckM ( Parks *et al.*, 2015) was used to estimate completeness and contamination, and GTDB-Tk ( Chaumeil *et al.*, 2022) was used for taxonomic classification.

## Genome sequence report

### Sequence data

**Figure 2.**
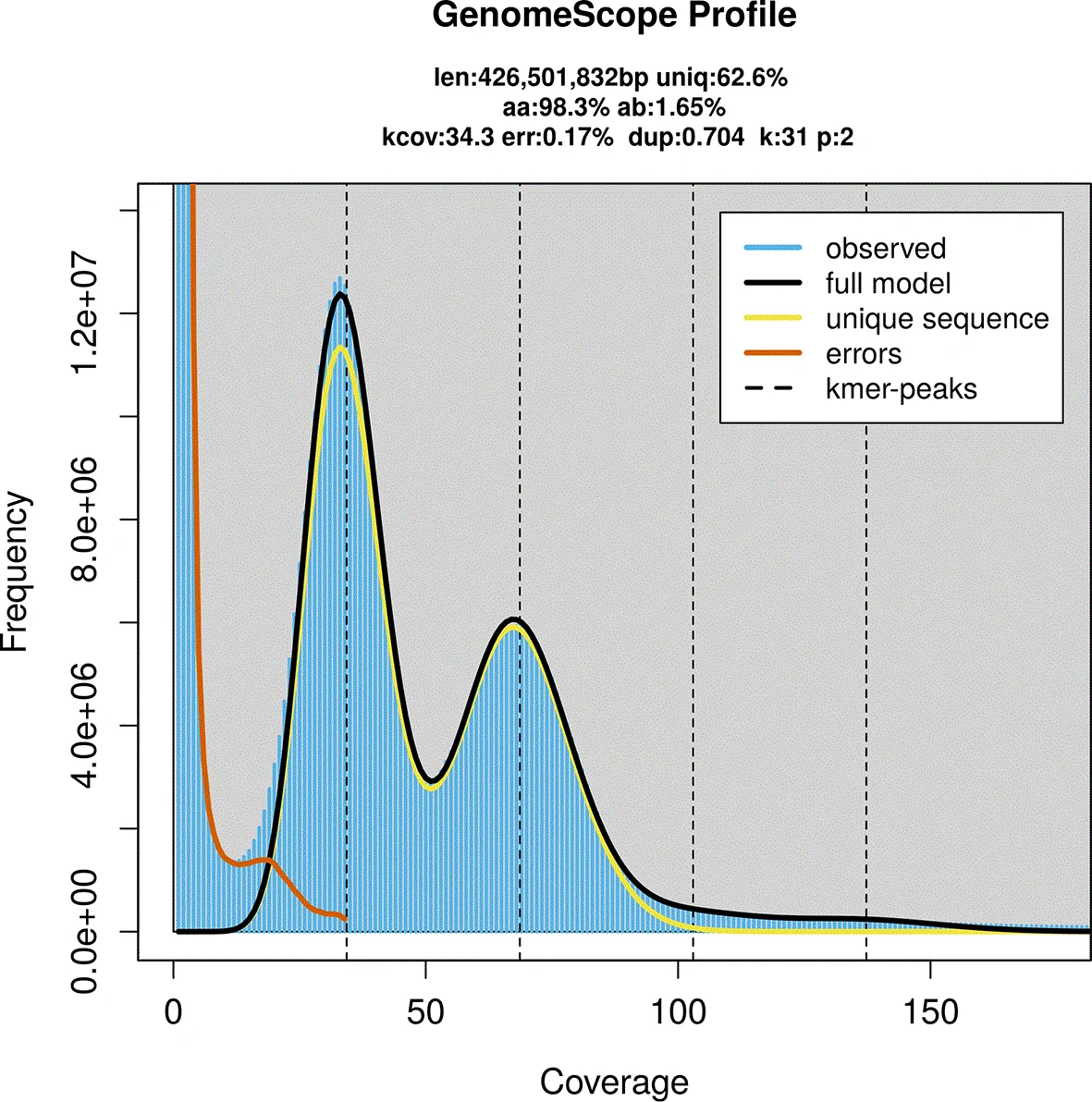
Frequency distribution of *k*-mers generated using GenomeScope2. The plot shows observed and modelled *k*-mer spectra, providing estimates of genome size, heterozygosity, and repeat content based on unassembled sequencing reads.

PacBio sequencing of the *Acropora cf. manni* specimen generated 32.35 Gb (gigabases) from 5.82 million reads, which were used to assemble the genome. GenomeScope2.0 analysis estimated the haploid genome size at 427.17 Mb, with a heterozygosity of 1.65% and repeat content of 37.42% ( Figure 2). These estimates guided expectations for the assembly. Based on the estimated genome size, the sequencing data provided approximately 69× coverage. Hi-C sequencing produced 142.57 Gb from 472.07 million reads, which were used to scaffold the assembly. RNA sequencing data were also generated and are available in public sequence repositories. Table 1 summarises the specimen and sequencing details.

**Table 1.** Specimen and sequencing data for *Acropora cf. manni* (BioProject PRJEB83136).

Platform	PacBio HiFi	Hi-C	RNA-seq
**ToLID**	jaAcrPulc1	jaAcrPulc1	jaAcrPulc1
**Specimen ID**	UDUK0000426	UDUK0000426	UDUK0000426
**BioSample (source individual)**	SAMEA115672038	SAMEA115672038	SAMEA115672038
**BioSample (tissue)**	SAMEA115672048	SAMEA115672047	SAMEA115672047
**Tissue**	modular colony	modular colony	modular colony
**Instrument**	Revio	Illumina NovaSeq X	Illumina NovaSeq X
**Run accessions**	ERR14048077	ERR14056184	ERR15140919
**Read count total**	5.82 million reads	472.07 million read pairs	58.95 million read pairs
**Base count total**	32.35 Gb	142.57 Gb	17.80 Gb

### Assembly statistics

The genome was assembled into two haplotypes using Hi-C phasing. Haplotype 1 was curated to chromosome level, while haplotype 2 was assembled to scaffold level. The final assembly has a total length of 459.26 Mb in 1 339 scaffolds, with 1 621 gaps, and a scaffold N50 of 30.84 Mb ( Table 2).

**Table 2.** Genome assembly statistics for *Acropora cf. manni.*

Genome assembly	Haplotype 1	Haplotype 2
**Assembly name**	jaAcrPulc1.hap1.1	jaAcrPulc1.hap2.1
**Assembly accession**	GCA_965118205.1	GCA_965118215.1
**Assembly level**	chromosome	scaffold
**Span (Mb)**	459.26	489.30
**Number of chromosomes**	14	-
**Number of contigs**	2 960	2 790
**Contig N50**	0.4 Mb	0.41 Mb
**Number of scaffolds**	1 339	1 265
**Scaffold N50**	30.84 Mb	13.48 Mb
**Longest scaffold length (Mb)**	36.46	24.16
**Organelles**	Mitochondrial genome: 18.48 kb	-

The scaffold count follows the NCBI assembly statistics and excludes organelle assemblies, which are reported separately where present.

**Figure 3.**
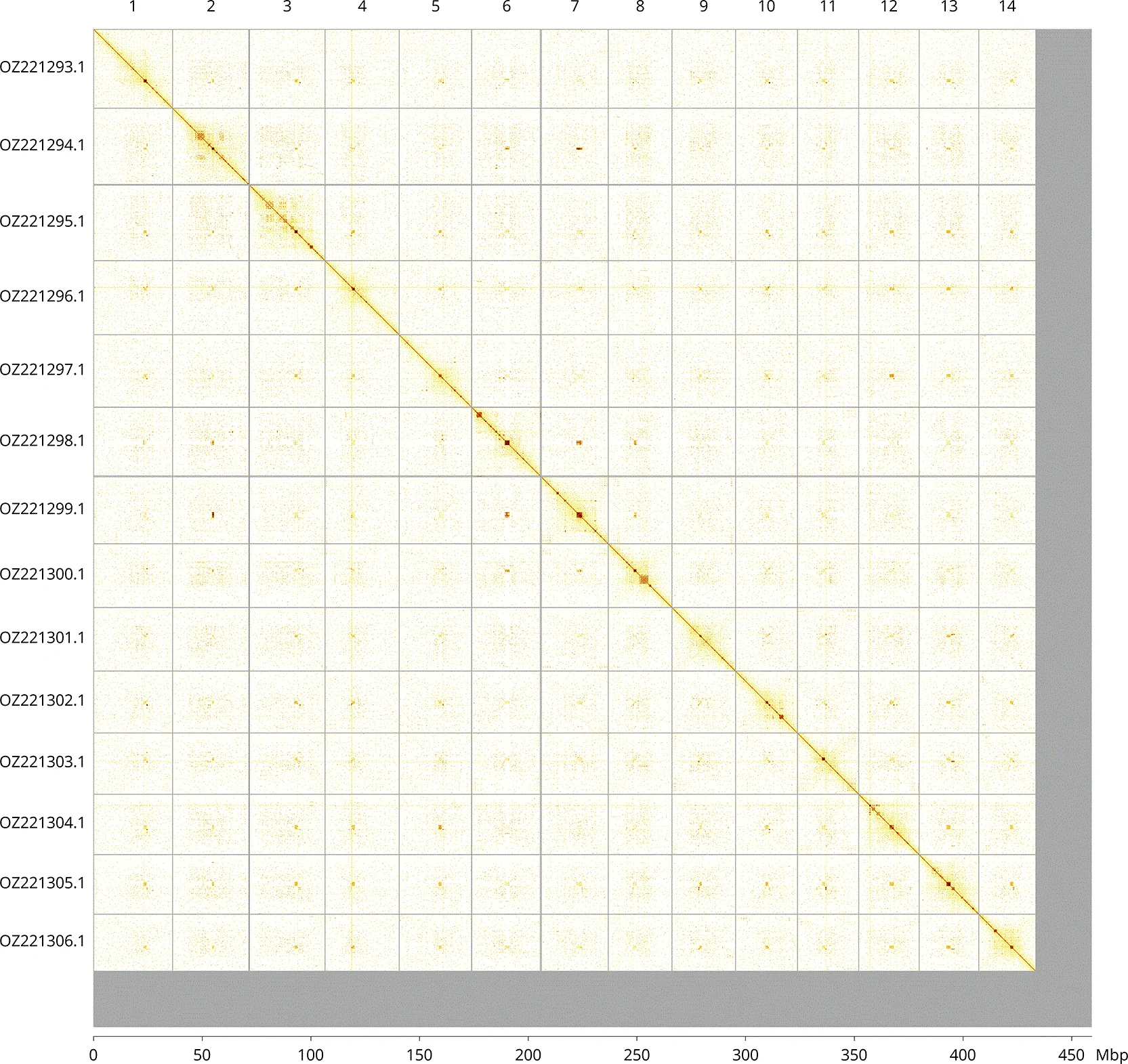
Hi-C contact map of the *Acropora cf. manni* genome assembly. Assembled chromosomes are shown in order of size and labelled along the axes, with a megabase scale shown below. The plot was generated using PretextSnapshot.

Most of the haplotype 1 assembly sequence (94.39%) was assigned to 14 chromosomal-level scaffolds. These chromosome-level scaffolds, confirmed by Hi-C data, are named according to size ( Figure 3; Table 3).

**Table 3.** Chromosomal pseudomolecules in the haplotype 1 genome assembly of *Acropora cf. manni* jaAcrPulc1.

INSDC accession	Molecule	Length (Mb)	GC%
OZ221293.1	1	36.46	39
OZ221294.1	2	35.38	39
OZ221295.1	3	34.73	39
OZ221296.1	4	34.15	39
OZ221297.1	5	33.39	39
OZ221298.1	6	31.95	39
OZ221299.1	7	30.84	39
OZ221300.1	8	29.31	39
OZ221301.1	9	29.21	39.50
OZ221302.1	10	28.55	39
OZ221303.1	11	28.06	39
OZ221304.1	12	27.88	39
OZ221305.1	13	27.37	39
OZ221306.1	14	26.22	39

The mitochondrial genome was also assembled (length 18.48 kb, OZ221307.1). This sequence is included as a contig in the multifasta file of the genome submission and as a standalone record.

### Assembly quality metrics

**Figure 4.**
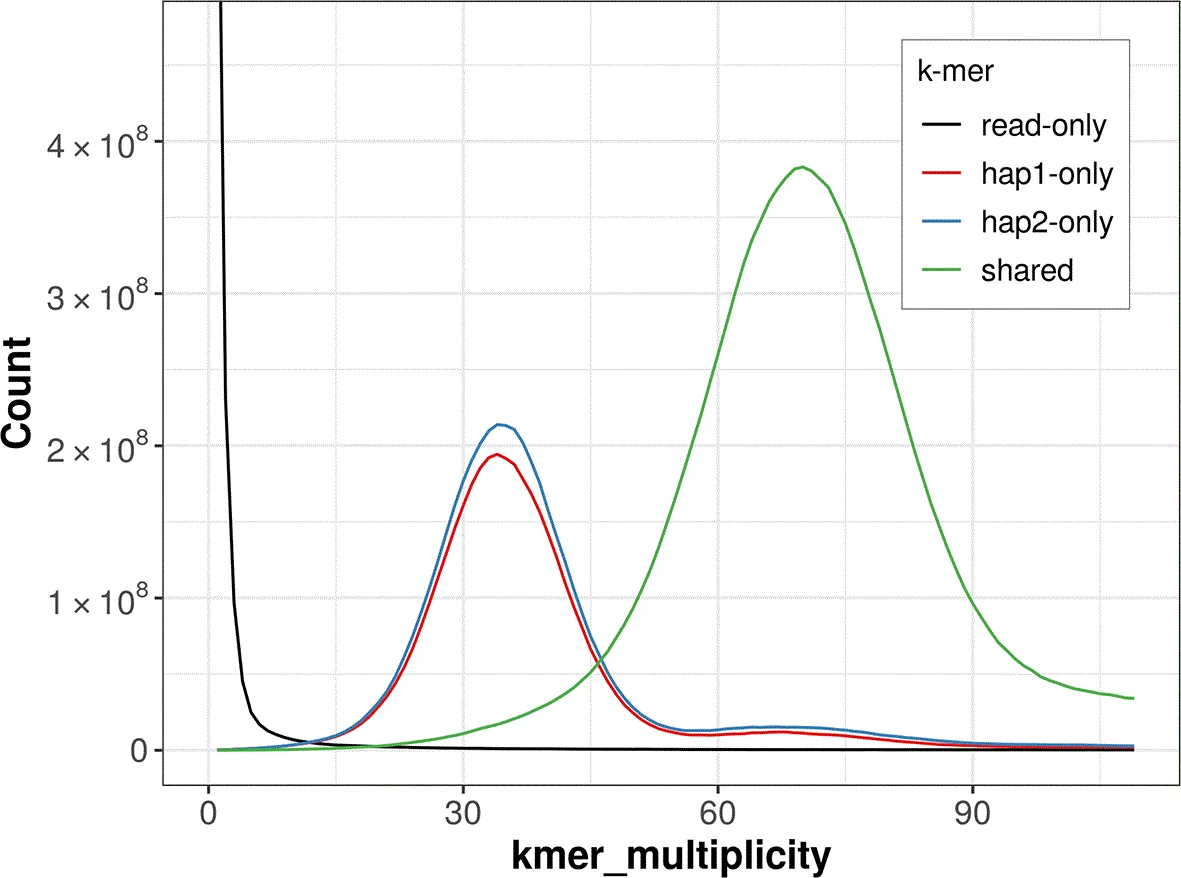
Evaluation of *k*-mer completeness using MerquryFK. This plot illustrates the recovery of *k*-mers from the original read data in the final assemblies. The horizontal axis represents *k*-mer multiplicity, and the vertical axis shows the number of *k*-mers. The black curve represents *k*-mers that appear in the reads but are not assembled. The green curve corresponds to *k*-mers shared by both haplotypes, and the red and blue curves show *k*-mers found only in one of the haplotypes.

For haplotype 1, the estimated QV is 60.2, and for haplotype 2, 61.4. When the two haplotypes are combined, the assembly achieves an estimated QV of 60.8. The *k*-mer completeness is 70.64% for haplotype 1, 73.76% for haplotype 2, and 99.25% for the combined haplotypes ( Figure 4).

**Figure 5.**
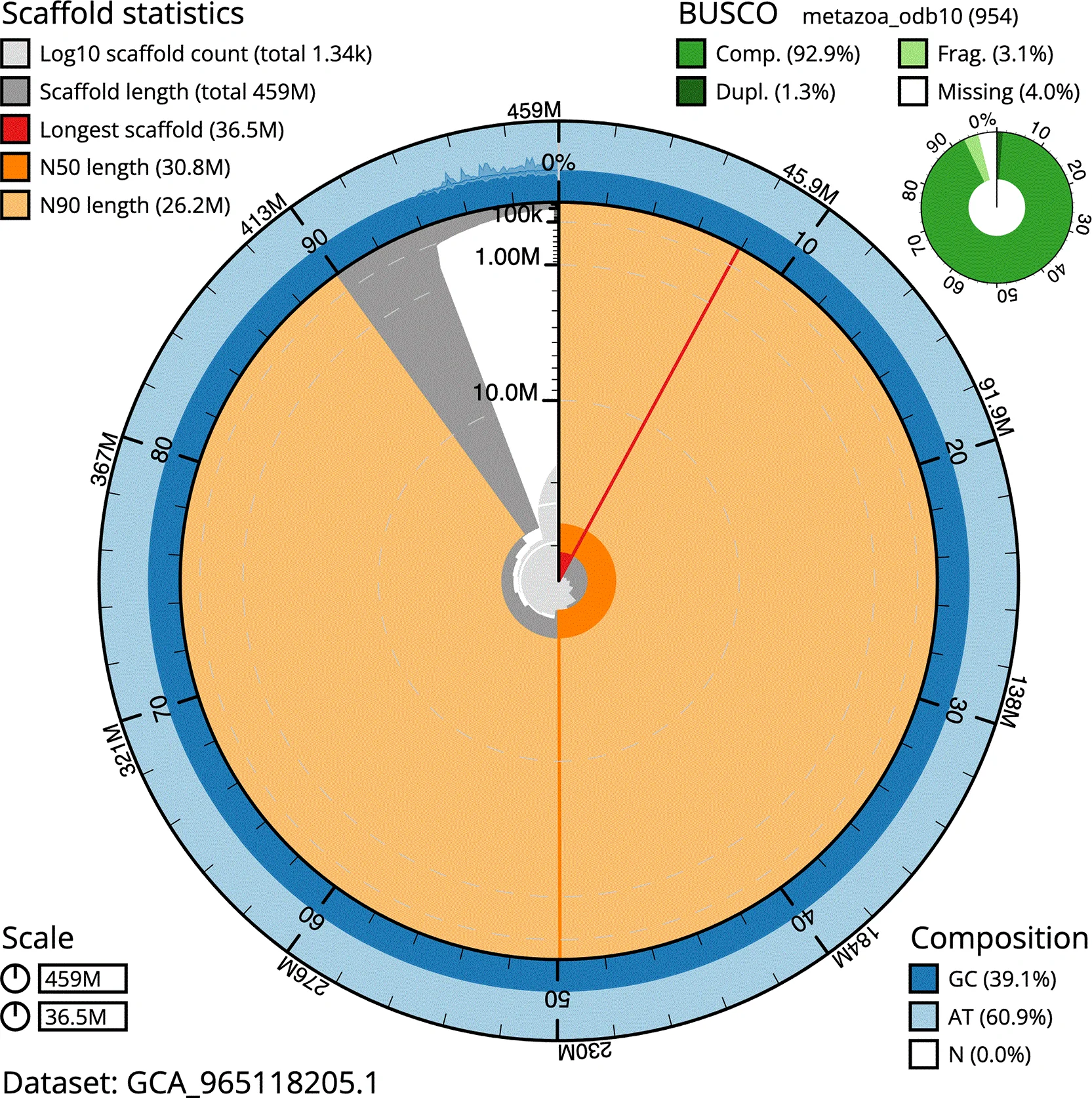
Assembly metrics for jaAcrPulc1.hap1.1. The BlobToolKit snail plot provides an overview of assembly metrics and BUSCO gene completeness. The circumference represents the length of the whole genome sequence, and the main plot is divided into 1 000 bins around the circumference. The outermost blue tracks display the distribution of GC, AT, and N percentages across the bins. Scaffolds are arranged clockwise from longest to shortest and are depicted in dark grey. The longest scaffold is indicated by the red arc, and the deeper orange and pale orange arcs represent the N50 and N90 lengths. A light grey spiral at the centre shows the cumulative scaffold count on a logarithmic scale. A summary of complete, fragmented, duplicated, and missing BUSCO genes in the set is presented at the top right. An interactive version of this figure can be accessed on the BlobToolKit viewer.

**Figure 6.**
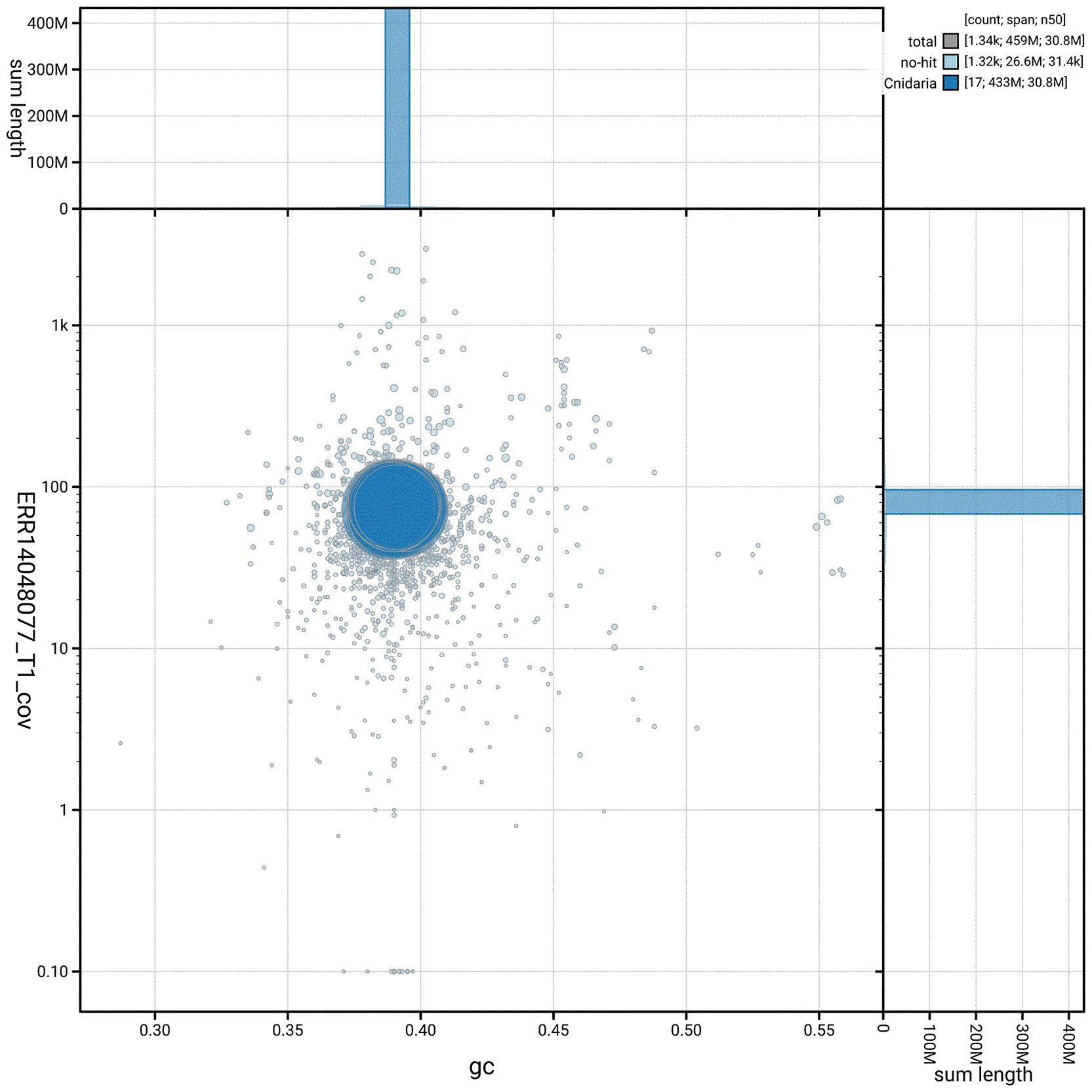
BlobToolKit blob plot for jaAcrPulc1.hap1.1. The plot shows base coverage (vertical axis) and GC content (horizontal axis). The circles represent scaffolds, with the size proportional to scaffold length and the colour representing phylum membership. The histograms along the axes display the total length of sequences distributed across different levels of coverage and GC content. An interactive version of this figure is available on the BlobToolKit viewer.

BUSCO analysis using the metazoa_odb10 reference set ( *n* = 954) identified 92.9% of the expected gene set (single = 91.6%, duplicated = 1.3%) in haplotype 1. For haplotype 2, BUSCO v.5.5.0 analysis identified 93.3% of the expected gene set (single = 91.8%, duplicated = 1.5%). The snail plot in Figure 5 summarises the scaffold length distribution and other assembly statistics for haplotype 1. The blob plot in Figure 6 shows the distribution of scaffolds by GC proportion and coverage for haplotype 1.

Table 4 lists the assembly metric benchmarks adapted from Rhie *et al.* (2021) and the Earth BioGenome Project Report on Assembly Standards January 2026. The EBP metric, calculated for haplotype 1, is **5.C.Q60**.

**Table 4.** Earth Biogenome Project summary metrics for the *Acropora cf. manni* assembly.

Measure	Value	Benchmark
EBP summary (haplotype 1)	5.C.Q60	5.C.Q40
Contig N50 length	0.40 Mb	≥ 0.1 Mb
Scaffold N50 length	30.84 Mb	= chromosome N50
Consensus quality (QV)	Haplotype 1: 60.2; haplotype 2: 61.4; combined: 60.8	≥ 40
*k*-mer completeness	Haplotype 1: 70.64%; Haplotype 2: 73.76%; combined: 99.25%	≥ 90%
BUSCO	Haplotype 1: C:92.9% [S:91.6%, D:1.3%], F:3.1%, M:4.0%, n:954; Haplotype 2: C:93.3% [S:91.8%, D:1.5%], F:3.4%, M:3.4%, n:954	S > 90%; D < 5%
Percentage of assembly assigned to chromosomes	94.39%	≥ 90%

*Notes:* The EBP summary uses log10(Contig N50); chromosome-level (C) or log10(Scaffold N50); Q (Merqury QV). BUSCO: C = complete; S = single-copy; D = duplicated; F = fragmented; M = missing; n = orthologues.

**Table 5.** Software versions and sources used for *Acropora cf. manni.*

Software	Version	Source
BLAST	2.14.0	https://ftp.ncbi.nlm.nih.gov/blast/executables/blast+/
BlobToolKit	4.3.9	https://github.com/blobtoolkit/blobtoolkit
BUSCO	5.5.0	https://gitlab.com/ezlab/busco
bwa-mem2	2.2.1	https://github.com/bwa-mem2/bwa-mem2
CheckM	2015-01-16	https://ecogenomics.github.io/CheckM/
DIAMOND	2.1.8	https://github.com/bbuchfink/diamond
fasta_windows	0.2.4	https://github.com/tolkit/fasta_windows
FastK	1.1	https://github.com/thegenemyers/FASTK
GenomeScope2.0	2.0.1	https://github.com/tbenavi1/genomescope2.0
Gfastats	1.3.6	https://github.com/vgl-hub/gfastats
GTDB-Tk	1.2.1	https://github.com/Ecogenomics/GTDBTk
Hifiasm	0.19.8-r603	https://github.com/chhylp123/hifiasm
HiGlass	1.13.4	https://github.com/higlass/higlass
MAGScoT	1.0.0	https://github.com/ikmb/MAGScoT
MerquryFK	1.1.0-c1	https://github.com/thegenemyers/MERQURY.FK
MetaMDBG	Pre-release	https://github.com/GaetanBenoitDev/metaMDBG
Minimap2	2.24-r1122	https://github.com/lh3/minimap2
MitoHiFi	3	https://github.com/marcelauliano/MitoHiFi
MultiQC	1.14; 1.17 and 1.18	https://github.com/MultiQC/MultiQC
Nextflow	23.10.0	https://github.com/nextflow-io/nextflow
Oatk	1.0	https://github.com/c-zhou/oatk
PretextSnapshot	0.0.5	https://github.com/sanger-tol/PretextSnapshot
PretextView	1.0.3	https://github.com/sanger-tol/PretextView
Prokka	1.14.5	https://github.com/tseemann/prokka
samtools	1.19.2	https://github.com/samtools/samtools
sanger-tol/ascc	0.1.0	https://github.com/sanger-tol/ascc
sanger-tol/blobtoolkit	0.6.0	https://github.com/sanger-tol/blobtoolkit
sanger-tol/curationpretext	1.4.2	https://github.com/sanger-tol/curationpretext
Seqtk	1.3	https://github.com/lh3/seqtk
Singularity	3.9.0	https://github.com/sylabs/singularity
TreeVal	1.4.0	https://github.com/sanger-tol/treeval
YaHS	1.2a.2	https://github.com/c-zhou/yahs

### Metagenome report

We recovered one bin from the metagenome assembly. The bin did not meet the criteria for a metagenome-assembled genome. It was assigned to the phylum Chlamydiota and had an estimated size of 1.32 Mbp, 85.5% completeness and 0.7% contamination.

## Author information

Contributors are listed at the following links:

• Members of the Wellcome Sanger Institute Tree of Life Management, Samples and Laboratory team
• Members of Wellcome Sanger Institute Scientific Operations – Sequencing Operations
• Members of the Wellcome Sanger Institute Tree of Life Core Informatics team
• Members of the EBI Aquatic Symbiosis Genomics Data Portal Team
• The Aquatic Symbiosis Genomics Project leadership

## Wellcome Sanger Institute – Legal and Governance

The materials that have contributed to this genome note have been supplied by a Tree of Life collaborator. The Wellcome Sanger Institute employs a process whereby due diligence is carried out proportionate to the nature of the materials themselves, and the circumstances under which they have been/are to be collected and provided for use. The purpose of this is to address and mitigate any potential legal and/or ethical implications of receipt and use of the materials as part of the research project, and to ensure that in doing so we align with best practice wherever possible.

The overarching areas of consideration are:

• Ethical review of provenance and sourcing of the material
• Legality of collection, transfer and use (national and international)

Each transfer of samples is undertaken according to a Research Collaboration Agreement or Material Transfer Agreement entered into by the Tree of Life collaborator, Genome Research Limited (operating as the Wellcome Sanger Institute) and in some circumstances other Tree of Life collaborators.

## Data Availability

European Nucleotide Archive: Acropora cf. manni. Accession number PRJEB83136; URL https://identifiers.org/ena.embl/PRJEB83136. The genome sequence is released openly for reuse. The *Acropora cf. manni* genome sequencing initiative is part of the Aquatic Symbiosis Genomics Project (PRJEB43743) and the Sanger Institute Tree of Life Programme (PRJEB43745). All raw sequence data and the assembly have been deposited in INSDC databases. The genome will be annotated using available RNA-Seq data and presented through the Ensembl pipeline at the European Bioinformatics Institute. Raw data and assembly accession identifiers are reported in Tables 1 and 2. Production code used in genome assembly at the WSI Tree of Life is available at https://github.com/sanger-tol . Table 5 lists software versions used in this study.
