# Supplementary material for: The chromosomal genome sequence of a staghorn coral, Acropora cf.  manni (Scleractinia: Acroporidae)
Source: Wellcome Open Res. 2026 Aug 27;11:551. [Version 1] doi: 10.12688/wellcomeopenres.27174.1 (PMC13570116; doi:10.12688/wellcomeopenres.27174.1)
Supplement: Peer review history

# Reviewer report: Reviewer response for version 1

**DOI:** 10.21956/wellcomeopenres.29913.r166030

**Published:** 2026-09-15

**Authors:** Nuria P Torres-Aguila, Biel Cassà

**Affiliations:** 1 Universitat de Barcelona, Barcelona, Spain

The Data Note submitted by D.J. Combosch et al. presents a newly assembled genome of the staghorn coral species *Acropora cf. manni*, with some insights into its associated microbiome. The manuscript is well structured and provides sufficient detail regarding the genome assembly. Nonetheless, I have identified few minor issues:

  

- **Figure1**: Panels are not described in the Figure legend. The different colours suggest that are different locations of the colony or different times. Please, describe relevant information of each panel in the figure legend, including if all the pictures belong to the same colony and which may be the sample acquired. Additionally, some pictures are of low quality, it would be useful if you can upload others with higher quality.
- **Methods section, Taxonomic information**: The information explained here is relevant due to the complex taxonomic history of the specie. Nonetheless, this subsection is not a standard Methods sections as it reports some unpublished results. I will suggest including the molecular result referred as supplemental file, if possible, as it seems relevant, and move the whole section inside “Sample acquisition”, as it is part of the sample description.
- **Methods section, Nuclei acid extraction**: “We used centrifuge-mediated fragmentation … for ultra-low input (ULI).” I suggest changing this phrase to impersonal to maintain coherence with the rest of the paragraph.
- **Methods section, PacBio HiFi library preparation and sequencing**: Molarity normalization of the libraries is not clear. Please unify the last paragraph with the last phrase of the previous paragraph. Additionally, the phrase ‘15 μL was used for making complexes.’ is ambiguous regarding to which complexes is referring to, please add few more details.
- **Genome sequence report section, Assembly statistics**: I suggest unifying the second paragraph with the first one.
- **Metagenome assembly and report**: The criteria used are not specified. Please include them in the assembly or in the report section.
- In the PDF version, most figures and tables appear before they are first cited in the text, making the article difficult to follow. Please review this.
- Nano **D**rop is referred as ‘Nano drop’. Please review the typo.
- Unify ‘μl’ to ‘μL’.

Overall, I consider this manuscript to be of interest to the scientific community and recommend its acceptance in Wellcome Open Research once the mentioned minor issues have been addressed.

Are sufficient details of methods and materials provided to allow replication by others?

Partly

Is the rationale for creating the dataset(s) clearly described?

Yes

Are the datasets clearly presented in a useable and accessible format?

Partly

Are the protocols appropriate and is the work technically sound?

Yes

Reviewer Expertise:

Transcriptomics, genomics, bioinformatics.

We confirm that we have read this submission and believe that we have an appropriate level of expertise to confirm that it is of an acceptable scientific standard, however we have significant reservations, as outlined above.

# Reviewer report: Reviewer response for version 1

**DOI:** 10.21956/wellcomeopenres.29913.r166033

**Published:** 2026-09-15

**Authors:** Bingpeng Xing

**Affiliations:** 1 Third Institute of Oceanography Ministry of Natural Resources, Xiamen, Fujian, China

This Data Note presents a high-quality, phased genome assembly of *Acropora cf. manni*. The assembly is highly complete, with strong QV and BUSCO scores, and most of haplotype 1 has been assigned to 14 chromosomal pseudomolecules, and the genomic resource will be valuable for coral taxonomy, population genomics, symbiosis research, and reef restoration.

Are sufficient details of methods and materials provided to allow replication by others?

Yes

Is the rationale for creating the dataset(s) clearly described?

Yes

Are the datasets clearly presented in a useable and accessible format?

Yes

Are the protocols appropriate and is the work technically sound?

Yes

Reviewer Expertise:

marine biodiversity，marine biology，genome，mangrove ecosystem

I confirm that I have read this submission and believe that I have an appropriate level of expertise to confirm that it is of an acceptable scientific standard.

# Reviewer report: Reviewer response for version 1

**DOI:** 10.21956/wellcomeopenres.29913.r166028

**Published:** 2026-09-11

**Authors:** Bernard M. Degnan

**Affiliations:** 1 The University of Queensland, Brisbane, Queensland, Australia

David Combosch and colleagues describe the chromosomal genome sequence of a staghorn coral, *Acropora* cf. *manni*. They provide sufficient background about this species of scleractinian coral and compelling rationale for the sequencing of genome from individuals inhabiting a coral reef from Guam, Mariana Islands. This genome will contribute to pursuits to resolve the evolution and biogeography of this species and, more broadly, the *Acropora* genus. The Methods and Genome Sequence Report are presented clearly and in sufficient detail.

Are sufficient details of methods and materials provided to allow replication by others?

Yes

Is the rationale for creating the dataset(s) clearly described?

Yes

Are the datasets clearly presented in a useable and accessible format?

Yes

Are the protocols appropriate and is the work technically sound?

Yes

Reviewer Expertise:

My expertise is in marine biology and genomics.

I confirm that I have read this submission and believe that I have an appropriate level of expertise to confirm that it is of an acceptable scientific standard.

# Reviewer report: Reviewer response for version 1

**DOI:** 10.21956/wellcomeopenres.29913.r167041

**Published:** 2026-09-08

**Authors:** Jayan Duminda M Senevirathna

**Affiliations:** 1 Uva Wellassa University, Badulla, Uva Province, Sri Lanka

This is a very interesting and potentially valuable study on the coral genome, and the genomic procedures appear to be comprehensive and technically robust. The BUSCO results are particularly encouraging, with 92.9% completeness for haplotype 1 and 93.3% for haplotype 2 using the *metazoa_odb10* reference set ( *n* = 954). The high proportions of single-copy BUSCOs (91.6% and 91.8%, respectively) and relatively low levels of duplication (1.3% and 1.5%, respectively) provide strong evidence for a high level of gene-space completeness in both haplotypes.

 However, an important issue that requires further consideration is the difference in the assembly levels of the two haplotypes. Although haplotypes 1 and 2 show very similar BUSCO completeness, haplotype 1 has been assembled to the chromosome level, whereas haplotype 2 remains at the scaffold level. BUSCO completeness primarily reflects the recovery of expected conserved genes and therefore does not necessarily indicate comparable chromosome-level contiguity, structural completeness, or assembly continuity. Consequently, the similar BUSCO scores should not be interpreted as evidence that the two haplotypes are equivalent in overall assembly quality.

 The authors should provide a clear explanation for why haplotype 2 could not be resolved to the chromosome level despite having a comparable BUSCO completeness.

 The manuscript should also discuss the potential implications of the scaffold-level status of haplotype 2 for downstream analyses, particularly those involving chromosome structure, synteny, gene organization, and comparative genomics.

Are sufficient details of methods and materials provided to allow replication by others?

Yes

Is the rationale for creating the dataset(s) clearly described?

Yes

Are the datasets clearly presented in a useable and accessible format?

Yes

Are the protocols appropriate and is the work technically sound?

Yes

Reviewer Expertise:

Aquatic molecular biology and biotechnology

I confirm that I have read this submission and believe that I have an appropriate level of expertise to confirm that it is of an acceptable scientific standard.
